# A policy scoping review of how ‘Hospitals at Home’ can and could support people with dementia

**DOI:** 10.1093/ageing/afag229

**Published:** 2026-08-03

**Authors:** Anthony Zheng Wang, Anita Wong, Nathan Davies, Sevim Hodge, Elizabeth L Sampson, Victoria Vickerstaff, Kate Walters, Catherine J Evans, Mark James Rawle, Jane Ward, Simon Conroy, Bastien Genet, Claudia Cooper

**Affiliations:** Faculty of Medicine and Dentistry, Queen Mary University of London, London, UK; UCL Medical School, University College London, London, UK; Centre for Psychiatry and Mental Health, Queen Mary University of London Wolfson Institute of Population Health, London, UK; Centre for Psychiatry and Mental Health, Queen Mary University of London Wolfson Institute of Population Health, London, UK; Centre for Psychiatry and Mental Health, Queen Mary University of London Wolfson Institute of Population Health, London, UK; Centre for Psychiatry and Mental Health, Queen Mary University of London Wolfson Institute of Population Health, London, UK; Department of Primary Care and Population Health, University College London Research, London, UK; Department of Palliative Care, Policy and Rehabilitation, Cicely Saunders Institute, King's College London, London, UK; Institute of Health Informatics, University College London, London, UK; Department of Primary Care and Population Health, University College London Research, London, UK; Centre for Psychiatry and Mental Health, Queen Mary University of London Wolfson Institute of Population Health, London, UK; Centre for Psychiatry and Mental Health, Queen Mary University of London Wolfson Institute of Population Health, London, UK; Centre for Psychiatry and Mental Health, Queen Mary University of London Wolfson Institute of Population Health, London, UK

**Keywords:** dementia, hospital at home, virtual ward, older people

## Abstract

**Introduction:**

People with dementia are often hospitalised due to comorbidities and superimposed delirium and have poorer outcomes in hospital compared to people without dementia, especially among under-served groups. Hospital at Home (HaH) schemes could reduce these inequalities by facilitating care in a familiar home environment that offers similar outcomes to acute hospital care with lower risks of harms.

**Aims:**

We aimed to investigate how dementia and cognitive impairment are considered within English health policy documents informing HaH implementation; considering potential impact on health inequalities and implications for future policy development.

**Methods:**

We searched websites, including UK governmental, NHS, social care and professional organisation sources, from 2015; and reference lists of included documents. We thematically analysed documents.

**Results:**

We included 17 documents, comprising clinical guidelines (*n* = 5), government guidance (*n* = 4), policy papers (*n* = 3), service evaluations (*n* = 3), a strategy document (*n* = 1) and case study (*n* = 1). We developed three themes: (i) benefits of HaH for people with dementia, including more person-centred care and familiar treatment environments; (ii) how HaH can be inclusively designed for people with dementia, accommodating needs and mitigating concerns over digital exclusion and safety; and (iii) the critical role of family carers in enabling HaH, including potential carer burden.

**Discussion:**

HaH models have potential to reduce health inequalities for people with dementia, but current implementation policies risk reinforcing inequalities, particularly among those without digital proficiency or carer support. Future policies should drive consistent inclusive eligibility criteria and processes for ensuring continuity with primary care, dementia care as a HaH staff core competency.

## Key points

- English government policy guiding HaH implementation acknowledges that this service model brings opportunities to deliver more person-centred, hospital-level care to people with dementia.
- It also acknowledges the critical role for family carers in providing this and the potential for exacerbating carer burden.
- HaH models have potential to reduce health inequalities for people with dementia, but current implementation policies risk reinforcing existing inequalities, particularly among those without digital proficiency or carer support.
- Future policies should drive consistent inclusive eligibility criteria and processes for ensuring care continuity through primary care interfaces and develop dementia care into a core competency for HaH staff.

## Introduction

In the UK, approximately 900 000 people have dementia [1]. Given the importance of familiar environments to people with dementia [2] and poorer acute hospital outcomes [3], Hospital at Home (HaH) programmes (sometimes called virtual wards) that provide hospital-level care at home [4] could improve dementia care quality. By safely shifting care into familiar surroundings, HaH can support the ‘10 Year Health Plan for England’ implementation [5]*.*

Low digital literacy and dementia-related confusion and/or superimposed delirium may structurally exclude people with dementia from HaH programmes [6], as digital technologies for monitoring and communication are integral to many models [4]. As HaH programmes expand across the NHS, it is critical to consider equity of provision. People with dementia experience stark care inequalities relative to people without dementia [7]; within dementia populations, under-served groups have higher and more complex needs, but reduced access to appropriate treatments [8].

This review asks: how are dementia and cognitive impairment considered within English health policy documents with potential to influence HaH implementation? How might policies impact health inequalities? What are the implications for policy development?

## Methods

**Figure 1:**
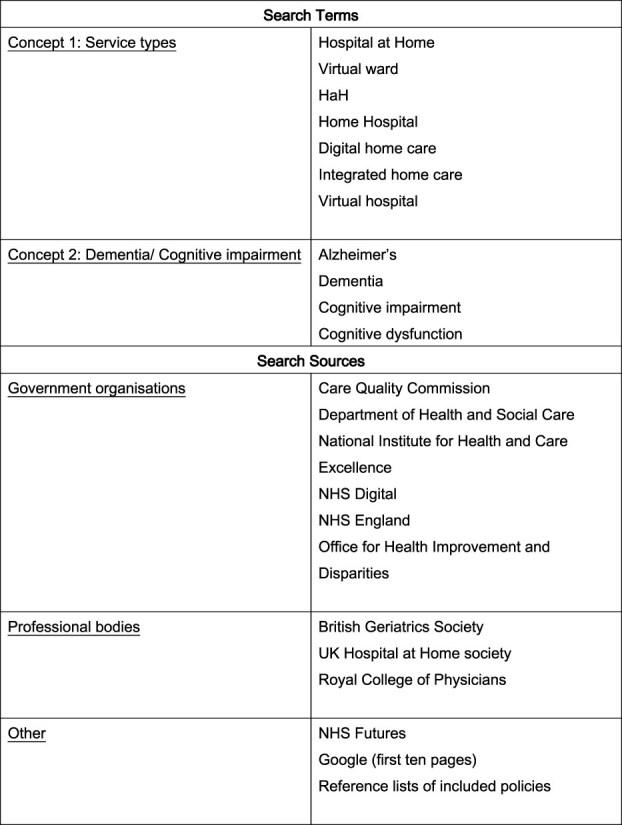
Search terms.

We conducted electronic searches for English health and social care policy documents published from 1 January 2015 until 26 August 2025. Using the search terms listed in Figure 1, A.Z.W. and A.W. independently searched UK Government, NHS England, professional and advocacy organisation websites, guided by Grey Matters guidelines, the Index of Grey Literature and Alternative Sources and Resources and searched Google using keywords (see Figure 1 for full list of sources). We searched reference lists, contacting authors for information when required, and used citation tracking via Google Scholar. We consulted expert clinicians and policy makers.

We included documents with potential to influence national, regional, local or clinical HaH implementation in populations with dementia and cognitive impairment. Where policies were superseded, we included the most recent version. We excluded NHS structural reform strategies and policies focused on people with learning disabilities, due to the distinct service models for these conditions.

A.Z.W. and A.W. reviewed potentially relevant documents against inclusion criteria, resolving discrepancies through discussion or involvement of CC.

### Data extraction and analysis

**Figure 2:**
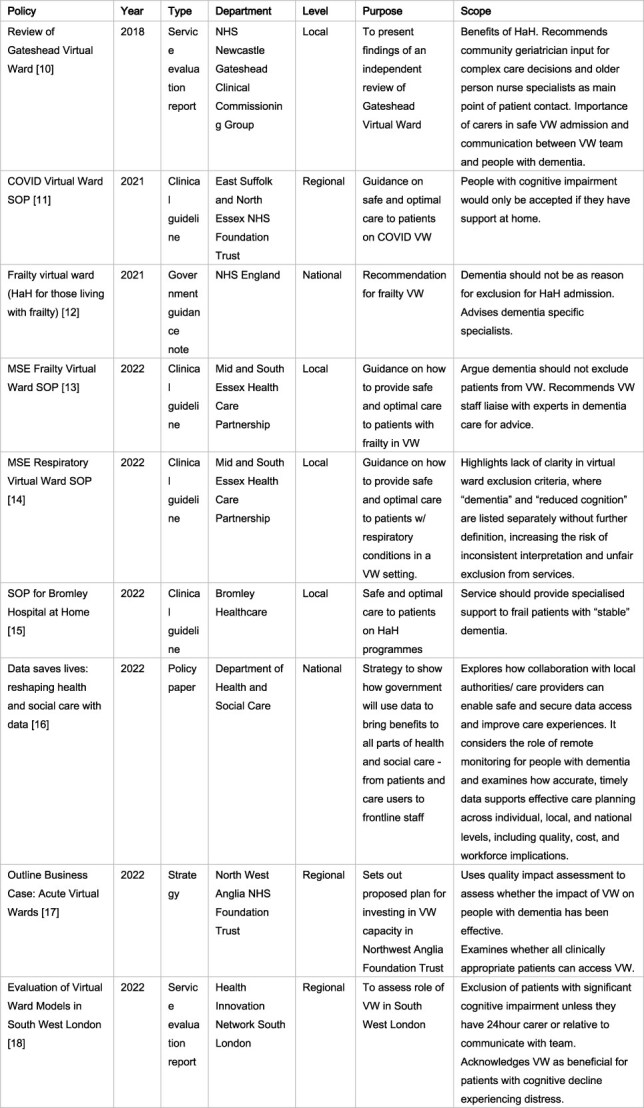

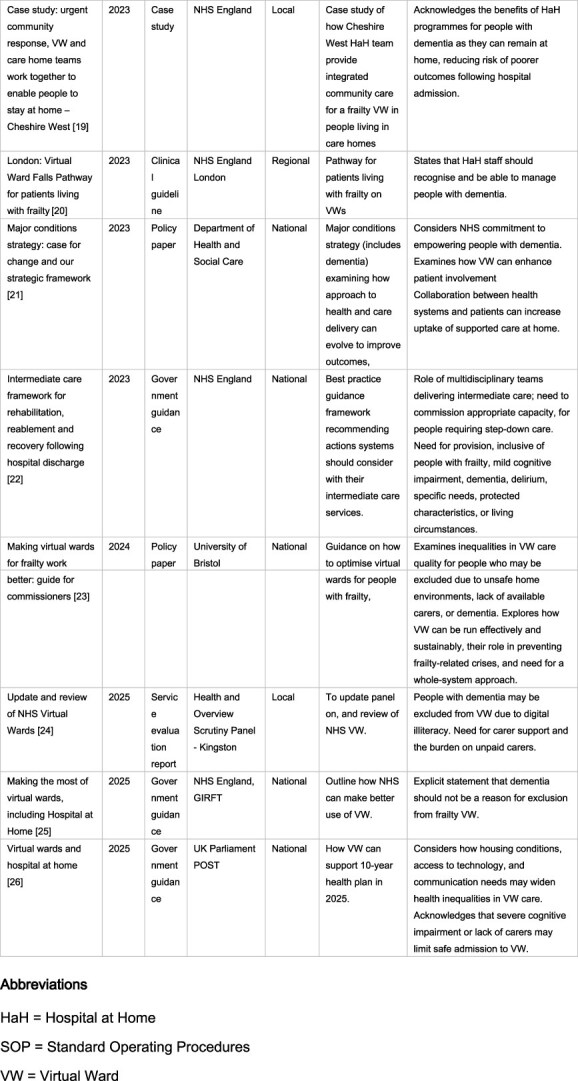
Description of the policy documents included in the review.

A.Z.W. and A.W. independently read all documents to identify potentially relevant sections, extracted descriptive data (Figure 2), then thematically analysed relevant sections, developing and combining codes which to create themes responding to review questions that the author group iteratively developed [9].

## Results

We included seventeen documents published from 2018 to 2025 (listed in Figure 2). They comprise policy papers (*n* = 3), case studies (*n* = 1), government guidance (*n* = 4), service evaluations (*n* = 3), guidelines (*n* = 5) and strategies (*n* = 1).

We developed three themes that cover the benefit of HaH for people with dementia, conflicting advice surrounding inclusion of people with dementia onto HaH and how they can be advocated for, and the importance of family carers in facilitating HaH care.

### Theme1: Potential benefits of HaH for people with dementia

This theme describes potential benefits of HaH for people with dementia including more patient-centred care and familiar treatment environments. Four out of six documents contributing to this theme outlined advantages of HaH for people with dementia in providing familiar care at home [10, 11] and own home environments [12, 13], and reducing anxiety and disorientation through avoiding hospital admissions [10–13]. Some discussed these advantages in terms of better clinical outcomes:

*“Anyone with cognitive difficulties can become very confused in a hospital environment, not knowing where they are. The virtual ward is particularly beneficial for these patients because, once they return home, the confusion tends to subside, allowing clinicians to better identify and manage the symptoms of the underlying condition”* [12].

Technological advances that detect changes in clinical status could enable better HaH outcomes in people with dementia less able to self-report symptoms [14, 15]:

*“Technologies like remote monitoring tools are also being used successfully to provide more targeted care, such as for individuals with dementia living at home, preventing or delaying escalating care needs”* [14].

The potential for digital records to streamline data sharing between care settings was noted to enable HaH models to provide person-centred care, by ‘improving the integration for those whose care spans health and social care, including many people with dementia’ [15].

### Theme 2: Inclusivity of HaH to people with cognitive impairment and dementia

This theme discusses conflicting advice around inclusion of people with dementia, and how staff competencies and digital infrastructure may enable or disable this. Five out of thirteen documents contributing to this theme advocated for including people with dementia in HaH [16–20], using ‘clinical judgement … where a person is living with dementia’ [17]. Government guidance states healthcare professionals should be ready to accommodate ‘people with particular conditions … to prevent exclusion or sub-optimal care’ [21]*.* No documents provided further guidance on HaH eligibility criteria in the dementia context, or how to accommodate their needs.

In contrast, five documents cited dementia or cognitive impairment as grounds for exclusion from HaH [12, 13, 22–24]. A Standard Operating Procedure for a respiratory virtual ward excluded ‘patient[s] with advanced dementia/frailty’ or ‘reduced cognition’ [23]. Concerns over ‘safety of [patients’] home environment [and] availability of carers’ [24] were discussed.

In three documents, HaH teams reported needing regular input from specialist clinicians to accommodate people with dementia. A 2018 HaH service evaluation recommended community geriatrician input for complex care decisions and older person nurse specialists for a ‘main point of contact for the family, care home and GP’ [10]; involving older people’s mental health teams was deemed ‘best practice’ for managing acute conditions in people with dementia [16, 17]. More recent documents reflected a shift to recommending all staff ‘regardless of profession, should have the skills and capabilities’ to manage frailty and dementia [25].

Two 2025 sources [13, 26] acknowledged barriers to inclusion of low levels of access to digital infrastructure such as ‘broadband connectivity, particularly in rural areas’ [13] in older populations at high dementia risk, and digital literacy among people with dementia*.* [26]

### Theme 3: The critical role of family carers

This theme described the critical enabling role of family carers. Six out of seven documents contributing to this theme [10, 12, 13, 22, 24, 26], commented that HaH access and eligibility depends on the involvement of formal or unpaid carers:

*“To be admitted to the virtual ward, patients … must not have significant cognitive impairment, unless they have a 24 hr carer or relative who is able to communicate with the team”* [12].

*“Patient exclusions: patients with cognitive impairment in **unsupported homes**”* [22].

Two sources considered HaH models unsuitable for those with dementia without ‘a carer or family member present’ [13, 26]; and 2025 government guidance identified carers as a facilitator for HaH safety for people with dementia:

*“Consider whether the patient would be safer in hospital rather than in a virtual ward, depending on … availability of carers, and the patient’s condition (e.g. people with delirium or dementia)”* [24].

Family and professional carers were seen as key to communication between people with dementia and the HaH team [10]: involvement of family members enabled a ‘more complete understanding’ of the individual’s needs and preferences [10].

Reliance on informal carers also raised concerns related to carer burden and equity for those without a family support network [26].

## Discussion

Current HaH policy documents recognise the benefits of providing hospital-level care in a familiar home setting for people with dementia. However, paradoxically, they were excluded from these services. Exclusion can be explicit, or implicit through eligibility requirements of digital proficiency or additional requirements for carer support [6]. This raises questions regarding criteria used to determine who is most likely to benefit from HaH care, which groups are excluded, and why.

We found few policy documents referencing HaH in dementia contexts, and none referencing challenges of superimposed delirium. People with dementia account for one in seven English hospital admissions [27] and have worse hospital outcomes [3], which HaH programmes can improve [4]. HaH admission can prevent delirium, which often drives hospital admission [28].

Many documents advocated for including people with dementia, but no further details were provided for how services and commissioners should operationalise this, nor regarding the interface with primary and community care, regarding how to support continuity of care on discharge.

Practical guidance could improve access to HaH services for people with dementia and increase equity of provision, encompassing inclusion criteria and workforce competencies. We noted a recent shift towards advocating for a workforce skilled to deliver dementia care within HaH, as opposed to expert liaison when required, aligning with national health and care workforce policy [5]. NHS England’s framework for digital inclusion advocates for maintaining non-digital healthcare support, alongside digital health approaches [29]; greater use of in-person HaH care models, valued by carers and older people [30], could increase equity.

We searched multiple national databases, but may have missed relevant policies. To mitigate this, we consulted expert clinicians and policy makers to develop and confirm the scope of our results.

## Conclusion

Future policies should drive consistent inclusive eligibility criteria and develop dementia care into a core competency for HaH staff. Addressing these gaps will support development of equitable HaH programmes and improve the quality of care provided to people with dementia.
